# Controlled Synthesis of Pt Doped SnO_2_ Mesoporous Hollow Nanospheres for Highly Selective and Rapidly Detection of 3-Hydroxy-2-Butanone Biomarker

**DOI:** 10.3389/fchem.2019.00843

**Published:** 2019-12-04

**Authors:** Haijie Cai, Haiquan Liu, Tianjun Ni, Yingjie Pan, Yong Zhao, Yongheng Zhu

**Affiliations:** ^1^College of Food Science and Technology, Shanghai Ocean University, Shanghai, China; ^2^Laboratory of Quality & Safety Risk Assessment for Aquatic Products on Storage and Preservation (Shanghai), Ministry of Agriculture, Shanghai, China; ^3^Shanghai Engineering Research Center of Aquatic-Product Processing & Preservation, Shanghai, China; ^4^School of Basic Medicine, Xinxiang Medical University, Xinxiang, China

**Keywords:** 3-hydroxy-2-butanone, gas sensor, Pt doped SnO_2_ nanomaterial, mesoporous hollow nanosphere, controlled synthesis

## Abstract

*Listeria monocytogenes (L. monocytogenes)* has been recognized as one of the extremely hazardous and potentially life-threatening food-borne pathogens, its real-time monitoring is of great importance to human health. Herein, a simple and effective method based on platinum sensitized tin dioxide semiconductor gas sensors has been proposed for selective and rapid detection of *L. monocytogenes*. Pt doped SnO_2_ nanospheres with particular mesoporous hollow structure have been synthesized successfully through a robust and template-free approach and used for the detection of 3-hydroxy-2-butanone biomarker of *L. monocytogenes*. The steady crystal structure, unique micromorphology, good monodispersit, and large specific surface area of the obtained materials have been confirmed by X-ray diffraction (XRD), Raman spectroscopy, Scanning Electron Microscopy (SEM), Transmission Electron Microscopy (TEM), X-ray Photoelectron Spectroscopy (XPS), Brunauer-Emmett-Teller (BET), and Photoluminescence spectra (PL). Pt doped SnO_2_ mesoporous hollow nanosphere sensors reach the maximum response of 3-hydroxy-2-butanone at 250°C. Remarkably, sensors based on SnO_2_ mesoporous hollow nanospheres with 0.16 wt% Pt dopant exhibit excellent sensitivity (R_air_/R_gas_ = 48.69) and short response/recovery time (11/20 s, respectively) to 10 ppm 3-hydroxy-2-butanone at the optimum working temperature. Moreover, 0.16 wt% Pt doped SnO_2_ gas sensors also present particularly low limit of detection (LOD = 0.5 ppm), superb long-term stability and prominent selectivity to 3-hydroxy-2-butanone. Such a gas sensor with high sensing performance foresees its tremendous application prospects for accurate and efficient detection of foodborne pathogens for the food security and public health.

**Graphical Abstract:**
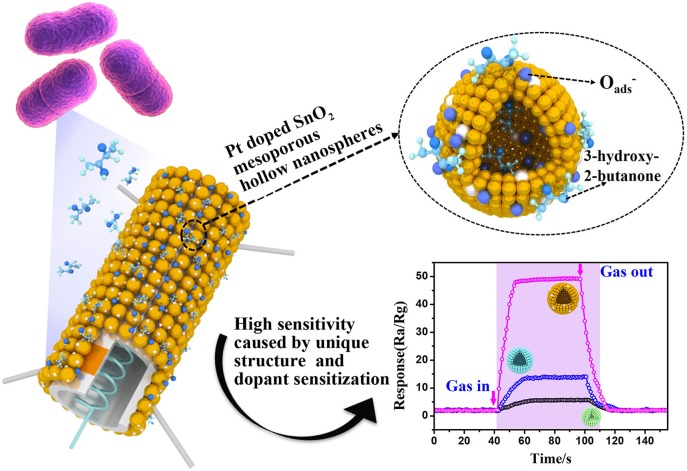
Pt doped SnO_2_ material with mesoporous hollow nanospheres structure were synthesized and used as semiconducting sensing materials to sensitively and selectively detect of trace 3-hydroxy-2-butanone biomarker. The enhanced sensing performance can be attributed to the unique and uniform mesoporous hollow nanosphere structure and dopant sensitization.

## Introduction

Bacterial foodborne pathogens are widely spread and cause millions of cases of human illness every year around the world (Carlson et al., 2018). *Listeria monocytogenes* is a zoonotic pathogen with strong adaptability and can survive in the temperature between 3 and 45°C (Ciesielski et al., 1987) and pH varying from 4.4 to 9.6 (Farber et al., 1992), it frequently associated with outbreaks of foodborne illness via intaking contaminated foods (Radoshevich and Cossart, 2018). People infected by *L. monocytogenes* may suffer from serious diseases such as meningitis, septicemia and hyperthermia gastroenteritis, especially in susceptible populations, the mortality rate is as high as 30% (Cheng et al., 2014). Great efforts have been made in detecting *L. monocytogenes*. Conventional methods such as biochemical tests and cell culture, are standard monitoring methods, but they are time-consuming and laborious in detection (Välimaa et al., 2015). In the contrast, smart detection of *L. monocytogenes*, such as immunological assay and molecular analysis, can significantly improve the detection efficiency, however, these methods suffer from either requiring professional technicians or complicated and expensive facilities (Zhao et al., 2018; Zhang Z. et al., 2019). Consequently, advances in rapid and facile examination techniques is of great significance to the economy and real-time monitoring of bacterial foodborne pathogens.

Gas sensor based on metal oxide semiconductor is deemed as a desirable tool for on-site inspection of gases by virtue of the advantages of simplicity, portability, cost-effective, easy-operation, and fast response to target gas molecules (Panahi et al., 2018). The wide variety of microbial volatile organic compounds that produced the proliferation of *L. monocytogenes* (Audrain et al., 2015; Wang Y. et al., 2016a) make it possible for metal oxide semiconductors gas sensors to be widely used in timely examination of biological hazards in food. Among these exhaled gases, 3-hydroxy-2-butanone is considered as a biomarker and can be used to indirectly identify and detect *L. monocytogenes* (Yu et al., 2015; Chen et al., 2017). Our previous research creatively used mesoporous WO_3_-based gas sensors to detect food-borne pathogens (Zhu et al., 2017). Later, Chen, Zhu et al. made new attempts in this direction and made further breakthroughs (Zhu et al., 2018; Chen et al., 2019). However, few studies have been conducted on the detection of *L. monocytogenes* by gas sensors based on metal sensitized nanomaterials. Accordingly, efforts are still needed to develop more sensitive and stable gas-sensitive nanomaterials for tracing *L. monocytogenes* in real time.

Tin dioxide (SnO_2_), a representative wide bandgap (3.6 eV) n-type semiconductor, offers great advantages in gas sensing owing to its quick response and good stability (Li Z. et al., 2016). However, the pure SnO_2_ based sensors are suffering from poor selectivity and harsh working temperature in gas detection. Two main promising approaches have been manifested to be most efficacious to address these issues: (1) controlling synthesis of novel and complex unique nanostructures; (2) doping or decorating with noble metals or metal oxide. In terms of structural control, the homogeneous mesoporous hollow nanostructure with large surface area and pore size can provide vast reaction sites for gasses access and effective pathways for rapid electronic transport, thus improving the sensitivity of gas sensors (Chen et al., 2011; Chen and Lou, 2013). As for element doping or decorating aspect, metal oxide semiconductors always fabricated and functionalized with noble metals like Pd, Au, and Rh (McFarland and Metiu, 2013; Hua et al., 2018), especially Pt (Wang et al., 2013, 2014), to improve the gas sensing performances. Xue's research group reported the synthesis of Pt doped SnO_2_ nanoflowers for highly sensitive gas sensor (Xue et al., 2019). Wang L. et al. (2016) synthesized hierarchical 3D SnO_2_ nanocomposites functionalized by Pt nanoparticles for sensitive and selective detection of ethanol. D'Arienzo et al. (2010) discussed the influence of catalytic activity on the response of Pt-Doped SnO_2_ gas sensors to reducing gas. Gas sensors based on Pt doped SnO_2_ enable high gas sensing performance, especially selectivity, due to the sensitization activity of the metals in improving the surface properties and adjusting the band structure (Li et al., 2014; Yao et al., 2014).

In this study, we present a low-cost and easy-to-use Pt doped SnO_2_ mesoporous hollow nanospheres based gas sensor for selective and rapid determination of 3-hydroxy-2-butanone biomarker. Firstly, the SnO_2_ mesoporous hollow nanospheres were controlled synthesized through a simple one-step templateless method, based on a classical inside-out Ostwald ripening mechanism. Then, Pt doped SnO_2_ compounds were obtained through a simple and novel approach possess by using dopamine as the adsorbent and reductant, and finally used to form the 3-hydroxy-2-butanone sensors. Compared to pure SnO_2_ mesoporous hollow nanospheres, the Pt doped SnO_2_ gas sensors achieve remarkably improved sensing performance toward 3-hydroxy-2-butanone vapor. Particularly, the 0.16 wt% Pt doped SnO_2_ mesoporous hollow nanospheres sensor display the highest sensitivity, reaching 48.69 (R_air_/R_gas_) toward 10 ppm 3-hydroxy-2-butanone at 250°C, while that of gas sensor assembled with pure SnO_2_ hollow nanospheres is only about 14.37 (R_air_/R_gas_). Moreover, this kind of gas sensor based on 0.16 wt% Pt sensitized metal oxide semiconductor presents fast response/recovery time (11/20 s, respectively), particularly low limit of detection (LOD = 0.5 ppm), excellent selectivity and long-term stability, showing greater advantages for rapid and ultrasensitive detection of *L. monocytogenes* in food, environment, clinical, and communal samples.

## Materials and Methods

### Chemical and Reagents

Ethanol solution (100%) and urea of AR grade were purchased from SinoPharm Chemical Reagent Co. Ltd. (Shanghai, China). Potassium stannate trihydrate (K_2_SnO_3_·3H_2_O, AR), dopamine hydrochloride, Tris-buffer (99.5%) and chloroplatinic acid (H_2_PtCl_6_·6H_2_O) were purchased from Sigma-Aldrich (St. Louis, MO, USA).

### Synthesis of SnO_2_ Mesoporous Hollow Nanospheres

According to the previous work (Lou et al., 2006), a robust and template-free approach has been taken for the controlled synthesis of SnO_2_ mesoporous hollow nanospheres. Typically, 1.15 mmol of K_2_SnO_3_·3H_2_O was dissolved into 60 mL of 37.5% ethanol-water bi-component solvent. After magnetic stirring for at least 5 min, a translucent solution with slightly white color was obtained. Thirty millimolars of urea was added into the solution before it was transferred to a 60 mL Teflon-lined autoclave. After reacting at 150°C for 24 h, the system was cooled down spontaneously. Finally, the white products were collected and washed with ethanol and deionized water for more than five times, then dried at 50°C overnight for further application.

### Doping of SnO_2_ Mesoporous Hollow Nanospheres With Pt Nanoparticles

Pt doped SnO_2_ mesoporous hollow spheres were fabricated through an *in-situ* reduction of the H_2_PtCl_6_**·**6H_2_O by using dopamine as the adsorbent and deoxidizer. One hundred milligrams of SnO_2_ mesoporous hollow nanospheres was evenly dispersed into the solution of 50 mg dopamine hydrochloride dissolved in 25 ml Tris-buffer (10 mM, pH 8.5). After stirring 12 h at room temperature, the gray product dopamine coated SnO_2_ mesoporous hollow nanospheres were washed with ethanol and water in turn and laid aside at 50°C overnight. Then, appropriate obtained product was added to 30 ml H_2_PtCl_6_·6H_2_O solution to reach the Pt dosage of 0.08 wt%, and keep stirring at normal temperature for 12 h. Afterwards, the white powder was obtained and then washed with ethanol and water and dried at 50°C overnight. Finally, the organic dopamine coating of Pt doped SnO_2_ mesoporous hollow nanospheres were removed by annealing at 500°C in air for 5 h, with an up/down ramp rate of 5°C/min. In addition to adjusting the concentration of tin sources, the above processes were repeated for preparation of others Pt doped SnO_2_ mesoporous hollow nanospheres at 0.12, 0.16, 0.24, and 0.48 wt% Pt loading.

### Materials Characterization

The morphology and crystalline structure of as-prepared gas sensing materials were explored by the followed methodologies. The structural characteristics were recorded by X-ray diffraction (XRD; Bruker, D8 Advance, Germany) with Cu-Kα radiation (λ = 0.15418 nm) in the range from 10° to 80° at normal temperature. The doping process of Pt metal after heat treatment was confirmed by Raman Spectrograph (Horiba, LabRAM HR Evolution, France) with an excitation wavelength of 532 nm. The microtopography of the materials was recorded with Scanning Electron Microscopy (SEM, FEI, Quanta FEG 450, USA) and Transmission Electron Microscope (TEM, FEI, Tecnai G220S-Twin, USA). The decorated Pt nanoparticles and their oxidized state were explored by X-ray Photoelectron Spectroscopy (XPS, Thermo Scientific, EscaLab 250Xi spectrometer). The specific surface areas of the particular mesoporous hollow nanostructures were calculated by Brunauer-Emmett-Teller (BET) method, using nitrogen as the adsorbate. Photoluminescence spectra (PL) of the Pt doped metal oxide have been acquired from a fluorescence spectrometer (Shimadzu International Trade Company, RF5301, Japan).

### Gas Sensor Fabrication and Measurement

The obtained nanomaterials were fully grounded with deionized water (4:1) to form a paste. The obtained homogeneous mixture was then carefully painted onto the ceramic tube welded with two gold electrodes and four platinum wires, which finally followed by sintering at 300°C for 2 h to remove the adhesive and get more closely combined gas sensor. Finally, a Ni-Cr heating resistance was plugged into the tube and then aged for 1 week under test conditions at 250°C to enhance the stability.

Gas sensing tests were performed on a commercial WS-30B Gas Sensing Measurement System developed and manufactured by Weisheng Instruments Co. (Zhengzhou, China). The test system comes with an 18 L test chamber. The gas sensing properties of the fabricated gas sensors to 3-hydroxy-2-butanone have been measured by recording the electrical resistance variation of the gas sensitive element and calculated according to the definition. The response value of the sensor is defined as the ratio of the resistances under target air and gas (S = R_air_/R_gas_), and the response and recovery time are defined as the time the sensor reaches 90% of the final equilibrium value after injection or release of the target gas.

## Result and Discussion

### Structural, Morphology, and Composition of the Samples

**Figure 1:**
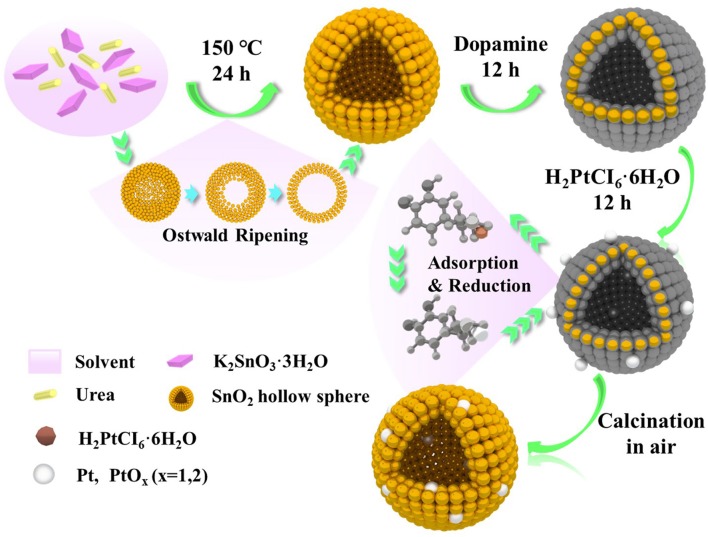
Schematic illustration of the formation of Pt doped SnO_2_ mesoporous hollow nanospheres via a simple template-free process.

Figure 1 illustrates the scheme of the controlled synthesize of SnO_2_ mesoporous hollow nanospheres doped with Pt metal. According to the Ostwald ripening mechanism, the controlled synthesis of SnO_2_ mesoporous hollow nanospheres was accomplished in the aqueous alcohol solution taking K_2_SnO_3_·3H_2_O as the precursor. Then, dopamine was used to form thin and surface-adherent polydopamine films on SnO_2_ hollow nanospheres. As shown in Figure 1, the bio-inspired dopamine molecule could spontaneously polymerize to the outer surface and interior of the SnO_2_ mesoporous hollow nanospheres. Subsequently, chloroplatinate adsorbed onto the positively charged amine groups of dopamine where they were reduced to platinum nanoparticles by dopamine (Bernsmann et al., 2011; Nda-Umar et al., 2018). Finally, Pt doped SnO_2_ mesoporous hollow nanospheres with good monodispersity, stable crystals and large BET surface area were obtained by calcination in air. Most of the platinum nanoparticles were oxidized to PtO_2_ during the calcination process. The use of dopamine as adhesive is not only simple and quick, but also inexpensive and “green” (Lee et al., 2007; Zhu et al., 2013). There is no need for any additional reducing agent, and is easily to remove the dopamine coating from the synthetic material through a calcination process in air.

**Figure 2:**
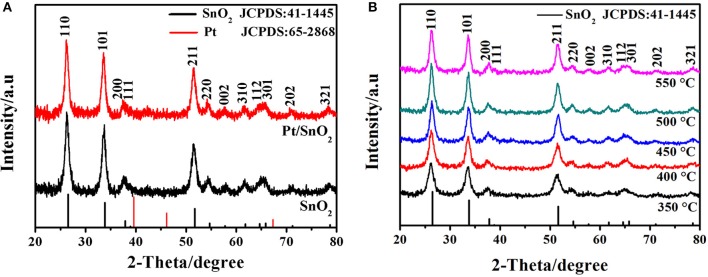
**(A)** X-ray diffraction patterns of as-synthesized pure and Pt doped SnO_2_ mesoporous hollow nanospheres samples obtained after calcination at 500°C in air. **(B)** XRD patterns of pure SnO_2_ mesoporous hollow nanospheres with the calcination temperature of 350, 400, 450, 500, and 550°C, respectively.

The crystal structures of the one-fold SnO_2_ and metal doped nano-sized mesoporous hollow spherical semiconductor materials were investigated by XRD analysis. The observed patterns in Figure 2A show that the three intense diffractions peaks at 2θ = 26.4°, 33.9°, and 51.8° correspond to (110), (101), and (211) planes of SnO_2_, respectively. Other diffraction peaks in Figure 2A are matched with the (200), (111), (220), (002), (310), (112), (301), (202), and (321) planes of SnO_2_. All of these emerged diffraction peaks are perfectly indexed to the JCPDS card No. 41-1445, confirming the tetragonal rutile crystal phase of the synthesized SnO_2_ nanomaterials. Meanwhile, no Pt nanoparticle peak of Pt doped SnO_2_ sample is observed, which is probably due to extremely small doses of added Pt (Ma et al., 2018). Since material synthesis is always carried out in a high temperature, the effect of temperature on the crystal structure is also worthy of attention (Wang et al., 2014). Therefore, the SnO_2_ were prepared at calcination temperatures varying from 350 to 550°C, and their XRD patterns were presented in Figure 2B. Obviously, every sample exhibit all of the characteristic diffraction peaks of SnO_2_, indicating the brilliant stable crystals of SnO_2_ mesoporous hollow nanospheres.

**Figure 3:**
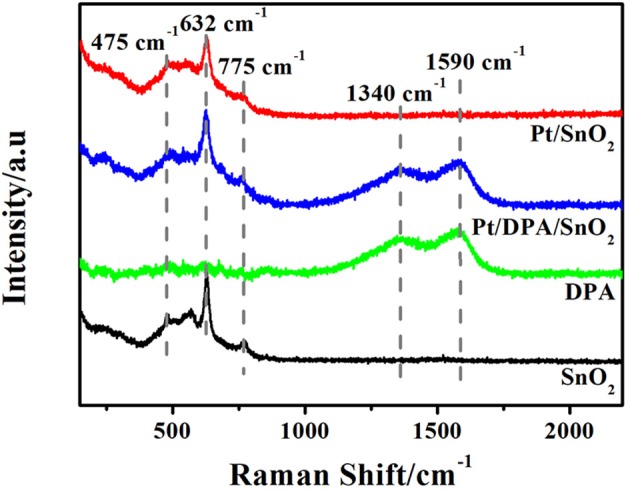
Raman spectra of as-synthesized products and intermediates (pure SnO_2_ mesoporous hollow nanosphere, pure DPA, Pt-DPA-SnO_2_ mesoporous hollow nanospheres and Pt doped SnO_2_ mesoporous hollow nanospheres).

Raman spectra of pristine SnO_2_, pure dopamine, Pt-DPA-SnO_2_ and Pt doped SnO_2_ are shown in Figure 3. The three strong peaks locate at 475 cm^−1^, 632 cm^−1^ and 775 cm^−1^ are attributed to E_g_, A_1g_, and B_2g_ vibrations of SnO_2_, respectively. As for intermediate products, two new fitted peaks appeared at 1,340 and 1,590 cm^−1^, representing the presence of dopamine. After calcining at 500°C for 5 h, no fingerprint peaks of dopamine in Pt doped SnO_2_ were observed, demonstrating the exhaustive removal of dopamine in the final product. Notably, none of the reflection peaks was related to Pt due to the extremely small size of well-dispersed Pt (Oh and Jeong, 2014).

**Figure 4:**
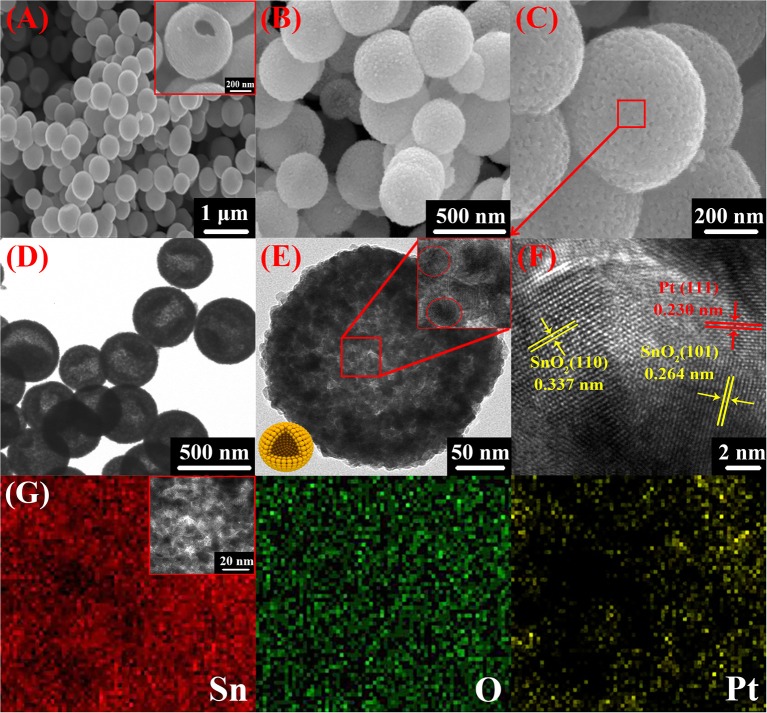
SEM **(A–C)**, TEM **(D)**, and HRTEM **(E,F)** of 0.16 wt% Pt doped SnO_2_ mesoporous hollow nanospheres; **(G)** elemental mappings of 0.16 wt% Pt doped SnO_2_ mesoporous hollow nanospheres for Sn, O, and Pt.

Figure 4 presents the morphological structure of Pt doped SnO_2_ nanomaterials. Apparently, the products consist of spherical hollow sphere with particle diameter of 400–500 nm and a shell thickness of ~30 nm. SEM micrographs (Figures 4A,B) display the spherical morphology of the nanomaterials with similar size distribution. The inset SEM image of Figure 4A and bright contour in TEM images (Figures 4D,E) clearly located in the center of the particle present the corresponding mesoporous hollow structure of the as-prepared Pt doped SnO_2_ nanomaterials. Figure 4D and the inset HRTEM image of Figure 4E reveal that the stable Pt doped SnO_2_ mesoporous hollow nanosphere as synthesized is composed of multiple layers of tin dioxide nanoparticles. The mesoporous hollow nanostructures stacked by multiple layers of SnO_2_ nanosphere have a highly usable alternating and stable structure (Wang Y. et al., 2016b), which is favorable for the diffusion of gases and then effectively improve the gas sensing performance. No phases of anchored Pt nanoparticles were observed in both SEM and HRTEM micrographs, possibly due to small amount of Pt catalyst (Bulemo et al., 2018). TEM mapping of Pt doped SnO_2_ nanomaterial was also conducted (Figure 4G), which notarize the existence and equidistributional of only Sn, O, and Pt component. Noting that the Sn and O are originated from the SnO_2_ hollow nanospheres, whereas Pt are exogenous doped.

**Figure 5:**
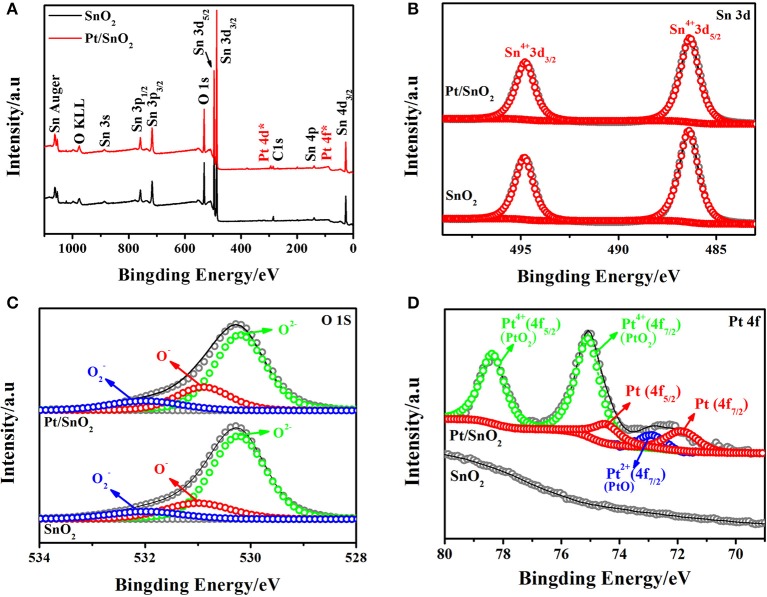
**(A)** Survey, **(B)** Sn 3d high resolution XPS spectrum of as-synthesized pure and Pt doped SnO_2_ mesoporous hollow nanospheres samples, **(C)** O 1s, **(D)** Pt 4f.

The surface chemical state of the semiconductor plays a non-negligible part in sensing properties. Therefore, the chemical states of the respective elements present in the singlet and Pt doped SnO_2_ mesoporous hollow nanospheres were analyzed by XPS. The complete spectra of the samples were displayed in Figure 5A. Apart from the C 1s calibration peak at 284.78 eV, only peaks fitted to Sn, O, and Pt are observed in Pt doped SnO_2_ samples, indicating the good monodispersity of the as-prepared Pt doped SnO_2_ samples. The signal decomposed into Sn 3d_5/2_ and Sn 3d_3/2_ (Figure 5B) two portions with peak located at 486.5 and 495.0 eV, respectively, which are typical characteristics of Sn^4+^ in tetragonal SnO_2_. No shifts of the Sn 3d peaks between the singular SnO_2_ and Pt doped SnO_2_ nanomaterials were observed mainly due to the low Pt dosage (Murata et al., 2013). The high-resolution XPS spectra of O 1s (Figure 5C) presents three peaks with binding energy at 530.2, 531.0, and 532.0 eV, which could be assigned to different chemical states of oxygen in the system: lattice oxygen (O^2−^) and absorbed oxygens (O^−^ and ${\text{O}}_{2}^{-}$), respectively (Jeong et al., 2018b). Usually, lattice oxygens are pretty stable and have no benefit in improving sensitivity, in the meantime, the absorbed oxygens are very active, which play a key role in gas sensitivity (Liu et al., 2015). As Figure 5D shows, Pt peaks were not detected in the pure SnO_2_ nanospheres. In contrast, five peaks were spited from XPS spectrum of Pt doped SnO_2_ samples (Jang et al., 2015; Bulemo et al., 2018). Two main peaks observed at 75.00 and 78.35 eV fitted to PtO_2_ (Kamble and Umarji, 2016), with a spin-orbit coupling energy between PtO_2_ 4f_7/2_ and PtO_2_ 4f_5/2_ of 3.35 eV. The peaks centered at 72.70 eV is suggested as assignable to PtO 4f_7/2_. The two peaks at 71.50 and 74.80 eV correspond to Pt 4f_7/2_ and Pt 4f_5/2_ (Kim et al., 2016). A large proportion of Pt nanoparticles were oxidized to form PtO_2_ at the annealing temperature ~500°C (Jang et al., 2015), caused the strong peaks of Pt^4+^, weak peaks of Pt and Pt^2+^.

**Figure 6:**
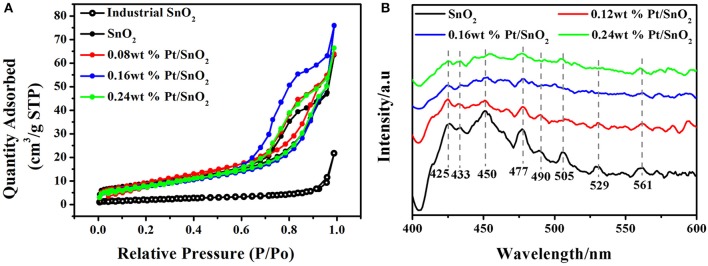
**(A)** N_2_ adsorption/desorption isotherms of commercial, pure SnO_2_ mesoporous hollow nanospheres and Pt doped SnO_2_ mesoporous hollow nanospheres. **(B)** PL spectra of as-synthesized pure SnO_2_ mesoporous hollow nanospheres and Pt doped SnO_2_ mesoporous hollow nanospheres.

To clearly investigate the surface adsorption properties of commercial, singular and Pt doped SnO_2_ mesoporous hollow nanospheres, we carried out BET test (Figure 6A) and Barrett-Joyner-Halenda (BJH) analysis (Table 1). Nitrogen adsorption-desorption isotherms of the pure and Pt doped SnO_2_ hollow nanospheres samples show typical type-IV curves with a hysteresis loop, demonstrating the uniform and large mesoporous structure of SnO_2_ hollow nanospheres. In the contrast, commercial SnO_2_ nanoparticles shows typical type-II curves. BET surface area of pure SnO_2_ mesoporous hollow nanospheres (28.2 m^2^/g STP) is nearly four-folds larger than commercial SnO_2_ (7.6 m^2^/g STP). Meanwhile, SnO_2_ mesoporous hollow nanospheres with different Pt doped dosages how different surface area and pore size. 0.08 wt% Pt doped SnO_2_ hollow nanospheres have the largest surface area (35.7 m^2^/g STP), while 0.16 wt% Pt doped SnO_2_ hollow nanosphere shows a little lower surface area of 31.5 m^2^/g STP but has the biggest pore volume (0.12 cm^3^/g) and pore size (15.4 nm) (Table 1). These pores with large size are conducive to the facilitating diffusion of gaseous molecules (Zhou et al., 2015), and the big pore volume can provide high-density of active surface locations (Zhang et al., 2016).

**Table 1:** BET Surface Area of as-synthesized pure and Pt doped SnO_2_ mesoporous hollow nanospheres.

**Sample**	**Commercial** ** SnO_**2**_**	**SnO_**2**_**	**0.08 wt%** ** Pt-SnO_**2**_**	**0.16 wt%** ** Pt-SnO_**2**_**	**0.24 wt%** ** Pt-SnO_**2**_**
BET surface area (m^2^/g)	7.6	28.2	35.7	31.5	29.7
Pore volume (cm^3^/g)	0.03	0.07	0.09	0.12	0.10
Pore size (nm)	18.1	9.4	10.2	15.4	12.7

Photoluminescence spectroscopy is a convenient and fast technique, which provides information about the types of oxygen vacancies in nanomaterials. PL emission spectra of the pure SnO_2_ present three strong peaks at 425, 450, and 477 nm, and five weak peaks at 433, 490, 505, 529, and 561 nm at an excitation wavelength of 385 nm (Figure 6B). According to the literatures, the purple luminescence peak (425 nm) can be attributed to the luminescence center formed by the tin gap or dangling bond (Arik et al., 2011), the PL peaks at 433, 450, and 477 nm can be ascribed to crystal defects in SnO_2_ matrix, the PL peaks appears at 490, 505, 529, and 561 nm which corresponds to green luminescence, and can be consider as singly charged oxygen vacancies in the material (Jean and Her, 2009). It is worth noting that the fluorescence intensity of Pt doped SnO_2_ is lower than pure SnO_2_, and the fluorescence intensity decreases gradually as the increases of platinum content, which can be attributed to the interaction of Pt metal and SnO_2_. Owing to the stronger ability to capture electronics of Pt than SnO_2_, the doped Pt can lead to reduction of donor type oxygen vacancies (Rani et al., 2007), thereby reducing the radiative recombination centers. Thus, the appearance of Pt and PtO_2_ not only occupy material voids, reduce pore volume and pore size (shown by BET results), but also decrease in the number of oxygen vacancies.

### Gas-Sensing Characteristics

Encouraged by the excellent structure of the synthetized SnO_2_ and Pt doped SnO_2_ nanomaterials, we further fabricated gas sensors based on commercial SnO_2_ (S_1_), pure SnO_2_ mesoporous hollow spheres (S_2_) and SnO_2_ mesoporous hollow nanospheres with different Pt doped content (S_3_–S_7_), respectively, to systematically explore their application prospect in the detection of *L. monocytogenes*. Commercial non-mesoporous SnO_2_ is employed as the reference for comparison. As previous studies investigated, the gas sensing characteristics depend upon the catalyst loading and dispersion. The insufficient loading of catalysts cannot reach the optimal catalytic effect, while, the excessive loading of catalysts on SnO_2_ mesoporous hollow spheres causes saturation and aggregation of catalysts, leading to a poor sensing performance. Hence, we carefully varied the dosages range of catalysts (S_3_: 0.08 wt%, S_4_: 0.12 wt%, S_5_: 0.16 wt%, S_6_: 0.24 wt%, S_7_: 0.48 wt%) to find the optimized dosage of catalysts.

**Figure 7:**
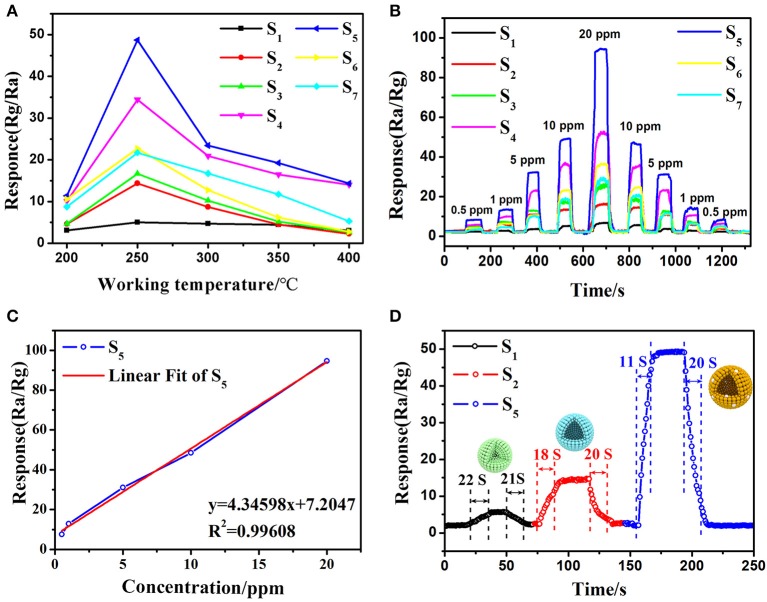
Typical response curve and variations of the sensors base on different samples: **(A)** response of the gas sensors S_1_-S_7_ versus different operating temperature (200–400°C) to 10 ppm 3-hydroxy-2-butanone, **(B)** dynamic 3-hydroxy-2-butanone (0.5–20 ppm) sensing transients of the gas sensors S_1_-S_7_, **(C)** the relationship between gas sensor S_5_ and concentration of 3-hydroxy-2-butanone gas and **(D)** response/recovery curves of gas sensor S_5_ (gas sensor S_1_ and S_2_ based on commercial SnO_2_ particles and as-synthesized pure SnO_2_ mesoporous hollow nanospheres, gas sensor S_3_-S_7_ based on 0.08, 0.12, 0.16, 0.24, 0.48 wt% Pt doped SnO_2_ mesoporous hollow nanospheres, respectively).

Basically, working temperature affects the adsorption and desorption characteristics of target analytes and the charge transport features on the surface of semiconductors, thus affects the gas sensing properties (Li et al., 2019). Therefore, the operating temperature tests of sensors based on commercial SnO_2_ particles, SnO_2_ mesoporous hollow spheres and Pt doped SnO_2_ mesoporous hollow spheres samples were firstly carried out in 10 ppm 3-hydroxy-2-butanone over temperatures ranging from 200 to 400°C (Figure 7A). The gas response gradually increases as the working temperature increase from 200 to 250°C, then decreases as the further elevation in operating temperature. Hence, 250°C is considered as the optimum working temperature for further sensing observations. At the same time, it is obvious that the Pt doped samples possess much higher sensitivity, especially sensor S_5_ based on 0.16 wt% Pt doped SnO_2_ mesoporous hollow nanospheres. The gas response toward 10 ppm 3-hydroxy-2-butanone is greatly enhanced from 14.37 to 48.69 by the decoration of Pt.

The dynamic gas sensor response of different pure and Pt loading amount SnO_2_ toward different concentrations of 3-hydroxy-2-butanone (0.5–20 ppm) an operating temperature of 250°C were then measured and shown in Figure 7B. The gas sensing results of all the fabricated gas sensors show increasing continuously with the increment of 3-hydroxy-2-butanone concentration while decrease as the concentration fall from 20 to 0.5 ppm, indicate the excellent reversibility and repeatability. The results of S_2_ sensor based on pure SnO_2_ (R_air_/R_gas_ = 14.37) to 10 ppm of 3-hydroxy-2-butanone is almost 3 times higher than S_1_ sensor based on commercial SnO_2_ (R_air_/R_gas_ = 5.04). Moreover, the gas sensor still has obvious response at 0.5 ppm 3-hydroxy-2-butanone concentration. Notably, the sensitivity can be increased by doping platinum. Among Pt decorated SnO_2_ sensors, the S_5_ sensor shows the best performance (R_air_/R_gas_ = 48.69, 10 ppm) and excellent linearity with the 3-hydroxy-2-butanone concentration (Figure 7C). According to the results of BET and PL tests, the highest response is mainly attributed to effect of platinum particles: the incorporation of Pt and Pt oxide inhibited the agglomeration of SnO_2_ particles and increased the specific surface area of the material then improves the sensitivity, however, excess Pt occupied the mesoporous space and even caused the decrease in oxygen vacancies, thereby reduced the response value (Singh and Singh, 2019). The response and recovery properties of sensors upon exposure to 10 ppm 3-hydroxy-2-butanone were also calculated from the sensing transients and the results were given in Figure 7D. The S_5_ sensor based on 0.16 wt% Pt sensitized SnO_2_ nanomaterials shows very low response (11 s) and recovery (20 s) times upon exposure to 3-hydroxy-2-butanone in those of commercial SnO_2_ (response/recovery: 22 s/21 s) and mesoporous hollow nanospheres (response/recovery: 18 s/20 s). The mesoporous hollow structure of SnO_2_ nanosphere with a high specific surface and large pore size can offer substantial active reaction sites for sensing test (Li Y. et al., 2016; Chen et al., 2018) and facilitate the quick and easy diffusion of gas molecules within the mesoporous structure in S_5_, resulting in the minimum response-recovery duration of S_5_ toward the target gas under the same conditions.

**Figure 8:**
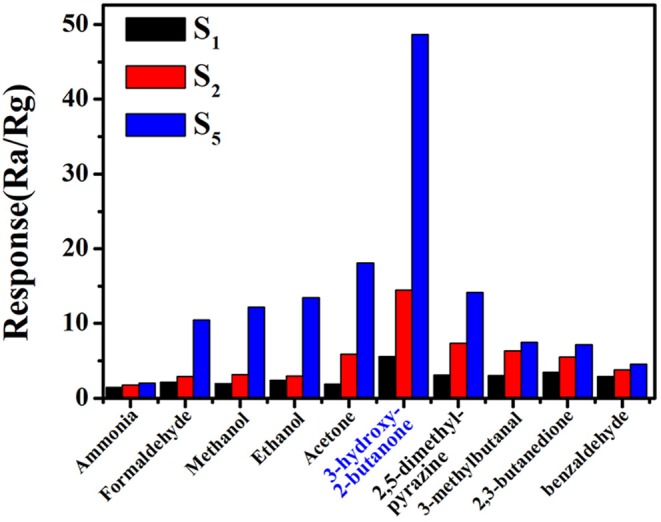
Selectivity of (gas sensor S_5_) 0.16 wt% Pt doped SnO_2_ based gas sensor toward 3-hydroxy-2-butanone at the optimal working temperature of 250°C.

Practically, the sensor with high sensitivity and fast response speed cannot satisfy the requirement of accurate and efficient detection of 3-hydroxy-2-butanone in complex gas environment. Therefore, we investigated the sensitivities of commercial, pure and 0.16 wt% Pt doped SnO_2_ mesoporous hollow nanospheres based sensors. The response of S_1_, S_2_, and S_5_ sensor in the presence of some common volatile organic compounds with a concentration of 10 ppm at 250°C, including acetone, ethanol, methanol, formaldehyde and ammonia, were shown in Figure 8. Obviously, sensor S_5_ based on 0.16 wt% Pt doped SnO_2_ mesoporous hollow nanospheres shows excellent selectivity to 3-hydroxy-2-butanone and less affected by other gases. Furthermore, four typical gases in exhaled *L. monocytogenes* breath, 2,3-butanedion, 3-methylbutanal, 2,5-dimethyl-pyrazine and benzaldehyde were also selected as interfering gases. The gas response of S_5_ toward 3-hydroxy-2-butanone is roughly 10.5, 6.8, 6.5, and 3.5 times higher than that toward benzaldehyde, 2,3-butanedione, 2,5-dimethyl-pyrazine and 3-methylbutanal, respectively. These results clearly show that the S_5_ sensor has a good selectivity to the exhaled 3-hydroxy-2-butanone of *L. monocytogenes*. The highly selective properties of sensor are mainly attribute to the sensitization effect of Pt. The formation of p-n junction caused by Pt element can effectively increase the amount of electron transfer and thus increase the response (Jang et al., 2015). Meanwhile, the Pt element are capable of dissociating hydroxyl group and keto group, leading to selective detection of 3-hydroxy-2-butanone (Wu et al., 2011; Jeong et al., 2018b). All results shown above demonstrate that the S_5_ gas sensor is suitable for the selective detection of the 3-hydroxy-2-butanone molecules in complex atmosphere.

**Figure 9:**
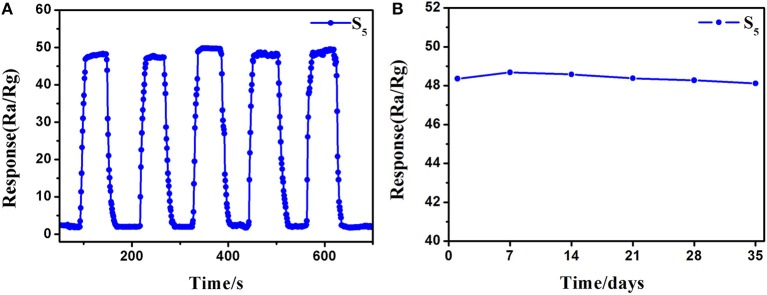
**(A)** Repeatability and **(B)** stability of (gas sensor S_5_) 0.16 wt% Pt doped SnO_2_ based gas sensor to 10 ppm 3-hydroxy-2-butanone at 250°C.

Excellent repeatability and long-term stability are also requisite in actual detection. The repeatability of the S_5_ sensor was recorded by exposing to 10 ppm of 3-hydroxy-2-butanone five times under the same conditions, and the response and recovery curves are shown in Figure 9A. The response level and response-recovery time in every test show no distinct difference, indicate that the as-fabricated 3-hydroxy-2-butanone sensor based on 0.16 wt% Pt doped SnO_2_ hollow nanospheres has good repeatability. The results of stability test on the S_5_ sensor to 10 ppm of 3-hydroxy-2-butanone show a neglectable change during the 5-week testing process (Figure 9B), reflecting the good long-term stability of 0.16 wt% Pt activated SnO_2_ sensors.

### Sensitization Mechanism of 3-Hydroxy-2-Butanone Sensing

**Figure 10:**
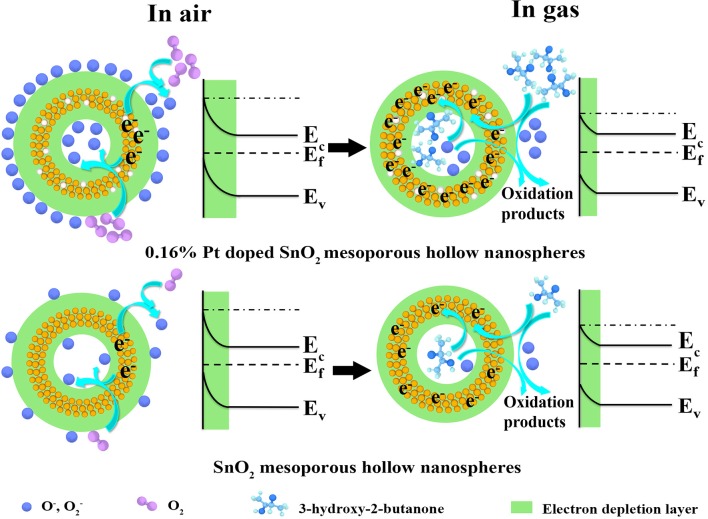
Schematic diagram of the proposed reaction mechanism of the (gas sensor S_2_) pure and (gas sensor S_5_) 0.16 wt% Pt doped SnO_2_ based gas sensor in air and 3-hydroxy-2-butanone.

On the basis of comprehensive analyses of experimental results, the enhanced sensing performances may be attributed to the unique mesoporous hollow nanosphere structure and the doped of Pt nanoparticles. The detection mechanism of the as-fabricated 3-hydroxy-2-butanone sensor is on account of the change in conductance of the semiconductor metal oxide nanomaterial when reacted with the target gas adsorbed on the sensing layer, which belongs to the surface-controlled mode (Wang L. et al., 2016). As shown in Figure 10, the schematic illustration presents the sensing mechanism and energy band levels of the pure and Pt doped SnO_2_ sensors. When the gas sensors are exposed to the air (left part of Figure 10), oxygen molecules are adsorbed on the SnO_2_ mesoporous hollow nanosphere and generate adsorbed oxygen ions (${\text{O}}_{2}^{-}$ and O^−^) by trapping electrons from the conduction band of SnO_2_ semiconductor, causing the formation of a thick electron depletion layer and low conductance of the sensor (Zhang D. et al., 2019). The chemical reactions involved in this process can be summarized as follows:

$$\begin{array}{c} \;O_{2}\to O_{2}\left(ads\right)\\ \;O_{2}\left(ads\right)+\;e^{-}\to {O_{2}}^{-}\left(ads\right)\;<150^{◦}\text{C}\\ {O_{2}}^{-}\left(ads\right)+\;e^{-}\to {2O}^{-}\left(ads\right)\;150-400^{◦}\text{C} \end{array}$$

As shown in the right part of Figure 10, once the target gas is injected, absorbed oxygens react with the 3-hydroxy-2-butanone molecules immediately and oxidize such reductive molecules to oxidation products. As a result, absorbed oxygens release electrons back to Pt doped SnO_2_ hollow nanospheres, leading to reduction of electron depletion layer and a low conductance (Yang et al., 2018).

On the basis of above theories, the improved sensing performance of the Pt doped SnO_2_ sensing materials can be concluded in two following aspects: (1) From the morphology structure aspect, the 0.16 wt% Pt doped SnO_2_ mesoporous hollow nanosphere with high specific surface area provides a mass of active sites both on the surface for oxygen molecules adsorb as well as sensitization agent to decorate and show their function (Hu et al., 2018). (2) As for Pt doping aspect, previous studies have shown that Pt nanocatalysts exist as oxidized forms (PtO_2_) at the annealing temperature ~500°C (Jang et al., 2015) which are p-type material, leading to the formation of p-n junction at the interface of SnO_2_ and PtO_2_ (Jeong et al., 2018a; Qiu et al., 2018). Therefore, this interaction between PtO_2_ and SnO_2_ play a significant role when the Pt doped SnO_2_ nanomaterials are exposed to 3-hydroxy-2-butanone, which expands the electron depletion region on SnO_2_ (Figure 10) and cause an increase in conductivity (Shao et al., 2016). Moreover, owing to the “spillover effect” of Pt element (Liu et al., 2017), oxygen molecules will be more easily absorbed on the surface of Pt and PtO_2_ compare with pristine SnO_2_ (Peng et al., 2018), leading to the promotion of sensing reaction between the tested gas molecules and adsorbed surface oxygen species. Under the above synergy between Pt dopant and SnO_2_, the conductivity of the Pt doped SnO_2_ gas sensor changes more greatly as the 3-hydroxy-2-butanone gases are in or out, leading to a higher response than the pristine SnO_2_. From all the above, the Pt doped SnO_2_ mesoporous hollow nanosphere sensor shows excellent 3-hydroxy-2-butanone sensing property.

## Conclusions

In summary, well-crystalline SnO_2_ mesoporous hollow spheres nanomaterials have been synthesized via a simple one-step template-free and robust method by using K_2_SnO_3_·3H_2_O as a precursor in the presence of precipitant urea, followed by doping with Pt by using H_2_PtCl_6_**·**6H_2_O as Pt source in the presence of reducing agent dopamine. The obtained 0.16 wt% Pt doped SnO_2_ mesoporous hollow nanospheres have high specific surface areas (31.5 m^2^/g) and large aperture of 15.3 nm. As a result of the doping of Pt element and the uniform mesoporous hollow structure with high surface areas, 0.16 wt% Pt doped SnO_2_ based sensors exhibit superior sensitivity, excellent long-term stability and highly selective detection to 3-hydroxy-2-butanone, at a wide range concentration. Specifically, it displays rapid response (response/recovery: 11 s/20 s), superior sensitivity (R_air_/R_gas_ = 48.69) toward low concentration of 3-hydroxy-2-butanone (10 ppm) at a working temperature of 250°C. Detailed analysis demonstrates that the improved 3-hydroxy-2-butanone sensing characteristics are possibly due to the “sensitization effect” driven by Pt nanoparticles platinum tungsten oxide. This 0.16 wt% Pt doped SnO_2_ mesoporous hollow nanosphere based gas sensor with superior 3-hydroxy-2-butanone sensitivity provide a great commitment for developing a novel, simple, accurate and rapid volatile organic compound portable sensor for the supervision of foodborne bacteria in food, environment, clinical, and communal samples.

## Data Availability Statement

All datasets generated for this study are included in the article/supplementary material.

## Author Contributions

YZhu and HC contributed to the experimental design. YP and YZha contributed to the data analysis and interpretation, manuscript writing, and manuscript revision. TN and HL contributed to the material synthesis and characterizations, and data acquisition. All authors reviewed the manuscript and approved the final version.

### Conflict of Interest

The authors declare that the research was conducted in the absence of any commercial or financial relationships that could be construed as a potential conflict of interest.
